# Balteatide: A Novel Antimicrobial Decapeptide from the Skin Secretion of the Purple-Sided Leaf Frog, *Phyllomedusa baltea*

**DOI:** 10.1155/2014/176214

**Published:** 2014-06-26

**Authors:** Lilin Ge, Xiaole Chen, Chengbang Ma, Mei Zhou, Xinping Xi, Lei Wang, Anwei Ding, Jinao Duan, Tianbao Chen, Chris Shaw

**Affiliations:** ^1^Natural Drug Discovery Group, School of Pharmacy, Queen's University, Belfast BT9 7BL, UK; ^2^School of Pharmacy, Fujian Medical University, Fuzhou, Fujian 350004, China; ^3^Jiangsu Key Laboratory for Traditional Formulae Research, Nanjing University of Chinese Medicine, Nanjing 210023, China

## Abstract

The skin secretions of Neotropical phyllomedusine leaf frogs have proven to be a rich source of biologically active peptides, including antimicrobials. The major families of antimicrobial peptides (AMPs) reported are the dermaseptins and phylloseptins and the minor families are the dermatoxins, phylloxins, plasticins, distinctins, and medusins. Here, we report a novel AMP of 10 amino acid residues (LRPAILVRIKamide), named balteatide, from the skin secretion of wild Peruvian purple-sided leaf frogs, *Phyllomedusa baltea*. Balteatide was found to exhibit a 90% sequence identity with sauvatide, a potent myotropic peptide from the skin secretion of *Phyllomedusa sauvagei*. However, despite both peptides exhibiting only a single amino acid difference (I/T at position 9), sauvatide is devoid of antimicrobial activity and balteatide is devoid of myotropic activity. Balteatide was found to have differential activity against the Gram-positive bacterium, *Staphylococcus aureus*; the Gram-negative bacterium, *Escherichia coli*; and the yeast, *Candida albicans*, and unusual for phyllomedusine frog skin AMPs, was most potent (MIC 32 mg/L) against the yeast. Balteatide was also devoid of haemolytic activity up to concentrations of 512 mg/L. Phyllomedusine frog skin secretions thus continue to provide novel AMPs, some of which may provide templates for the rational design of new classes of anti-infective therapeutics.

## 1. Introduction

The development of novel classes of anti-infective therapeutics has become an imperative within the pharmaceutical industry as pathogens continue to become resistant to all currently available antibiotics on a global scale [1–3]. Peptides, although generally shunned by the industry as drug candidates, have attracted much research attention as current evidence suggests that they are of ancient origin and occur as a component of front-line defence against microbial infection within the innate immune systems of both invertebrates and vertebrates and they even occur in plant tissues [2, 3].

More than 500 different antimicrobial peptides (AMPs) have been reported from diverse natural sources and the majority of those present in contemporary online databases are of amphibian skin origin [4]. One of the most important attributes of these amphibian skin AMPs is their broad spectrum of activity against bacteria, fungi, viruses, and protozoa [5, 6]. Structure/activity studies have revealed some common features of this class of peptides. Most are cationic and amphipathic containing a significant number of positively charged and hydrophobic amino acid residues; often the C-terminus is amidated effectively removing a membrane-repelling negative charge and replacing this with a hydrophobic membrane-interacting group; those that display antifungal activity often contain a high proportion of polar neutral amino acid residues [7–9]. The general mechanism of action of these AMPs is thought to involve initial electrostatic binding to the outer surfaces of cell membranes and then insertion of aggregates into the membranes to form pores through which the passage of ions and water is unregulated, leading to cell lysis [10]. Although there are major obstacles to the use of AMPs systemically, they appear to be effective for topical use as they have been shown to be capable of penetrating the stratum corneum and hence could be used to prevent infection of wounds [11]. The amphibians would appear to have evolved these AMPs for such a topical anti-infection role.

The Central and South American leaf frogs of the subfamily Phyllomedusinae have been found to possess some of the most molecularly complex and biologically potent skin secretions of any amphibians [12]. For these reasons and because of their species diversity and relative abundance, they have been widely studied for several decades [13]. Here, we report the presence of a novel AMP, named balteatide, from the skin secretions of wild specimens of the hitherto unstudied Peruvian species, the purple-sided leaf frog,* Phyllomedusa baltea. *Balteatide is a C-terminally amidated decapeptide, LRPAILVRIKamide, which displays particular potency against the pathogenic yeast,* Candida albicans*.

## 2. Methods and Materials

### 2.1. Acquisition of* Phyllomedusa baltea* Skin Secretion

Lyophilised skin secretion (40 mg dry weight), from wild Peruvian specimens of* Phyllomedusa baltea,* was obtained from PeruBiotech E.I.R.L. Skin secretions were obtained from the dorsal surfaces of frogs by gentle transdermal electrical stimulation. Briefly, the moistened skin was stimulated by platinum electrodes (6 V DC; 4 ms pulse-width; 50 Hz) for two periods of 20 s duration following which the secretion was washed from the skin using deionised water, snap-frozen in liquid nitrogen, and lyophilised. Lyophilisate was stored at −20°C prior to transportation and analysis.

### 2.2. Isolation and Structural Characterisation of Antimicrobial Activity from Lyophilised* P. baltea* Skin Secretion

A 5 mg sample of lyophilised* P. baltea* skin secretion was dissolved in 0.5 mL of trifluoroacetic acid (TFA)/water (0.05/99.95; v/v) and clarified by centrifugation (1100 ×g; 5 min). The supernatant was decanted and subjected to reverse-phase HPLC fractionation using a Waters HPLC system fitted with an analytical column (Phenomenex C-5; 250 mm × 4.6 mm). This was eluted with a linear gradient formed from TFA/water (0.05/99.95; v/v) to TFA/water/acetonitrile (0.05/19.95/80.0; v/v/v) in 240 min at a flow rate of 1 mL/min. The column effluent was continuously monitored spectrophotometrically at *λ* 214 nm and fractions (1 mL) were collected automatically at minute intervals. Samples (100 *μ*L) were removed from each fraction, lyophilised, and stored at −20°C prior to subjecting to antimicrobial assays. The molecular masses of peptides in each fraction displaying antimicrobial activity were analysed using matrix-assisted laser desorption/ionisation, time-of-flight mass spectrometry (MALDI-TOF MS) on a linear time-of-flight Voyager DE mass spectrometer (Perseptive Biosystems, MA, USA) in positive detection mode using *α*-cyano-4-hydroxycinnamic acid as the matrix. The amino acid sequence of the major resolved peptide in the antimicrobially active fraction was determined by MS/MS fragmentation sequencing using a LCQ-Fleet electrospray ion-trap mass spectrometer.

### 2.3. Antimicrobial Activity Screening Assays

Standard model nonpathogenic bacterial and yeast strains were used in these antimicrobial activity screening assays. Microorganisms used were the Gram-positive bacterium,* Staphylococcus aureus* (NCTC 10788); the Gram-negative bacterium,* Escherichia coli* (NCTC 10418); and the yeast,* Candida albicans* (NCPF 1467). The antimicrobial activity of peptides in reverse phase HPLC fractions of skin secretion was initially evaluated using inhibition zone assays on Luria-Bertani (LB) agarose plates [14]. To assess microbicidal effects, the lyophilised samples of each chromatographic fraction, following reconstitution in phosphate-buffered saline (PBS, pH 7.4 - 200 *μ*L), were added to separate 2 mm diameter holes that had been punched in the surface of the agar plate. The plates were then incubated at 37°C overnight.

### 2.4. Molecular Cloning of the Novel AMP Biosynthetic Precursor-Encoding cDNA from a* P. baltea* Skin Secretion-Derived cDNA Library

A second 5 mg sample of lyophilised* P. baltea* skin secretion was dissolved in 1 mL of cell lysis/mRNA protection buffer supplied by Dynal Biotech, UK, and polyadenylated mRNA was isolated by magnetic oligo-dT beads as described by the manufacturer (Dynal Biotech, UK). The isolated mRNA was subjected to 5′- and 3′-rapid amplification of cDNA ends (RACE) procedures to obtain full-length AMP biosynthetic precursor nucleic acid sequence data using a SMART-RACE kit (Clontech, UK) essentially as described by the manufacturer. Briefly, the 3′-RACE reactions employed a nested universal (NUP) primer (supplied with the kit) and a sense primer (S: 5′-TIMGICCIGCIATHYTIGT-3′) (I = deoxyinosine, M = A/C, H = A/T/C, and Y = C/T) that was complementary to the N-terminal amino acid sequence, L/I-R-P-A-L/I-L/I-V-, of the N-terminal region of balteatide. The 3′-RACE reactions were purified and cloned using a pGEM-T vector system (Promega Corporation) and sequenced using an ABI 3100 automated sequencer. The sequence data obtained from these 3′-RACE products were used to design a specific antisense primer (AS: 5′-GTGCTCCTCAGAGCTATGACTT-3′) to a conserved site within the 3′-nontranslated region of the balteatide-encoding transcript. 5′-RACE was carried out using this specific primer in conjunction with the NUP RACE primer and resultant products were purified, cloned, and sequenced.

### 2.5. Peptide Synthesis and Purification

Both balteatide and its structural homologue, sauvatide, from* Phyllomedusa sauvagei* skin secretion [15], were synthesised by solid phase methodology using Rink amide resin and standard Fmoc chemistry, by means of an automated PS3 peptide synthesiser (Protein Technologies, USA), followed by deprotection and cleavage from the resin. Each synthetic peptide was analysed by both reverse phase HPLC and MALDI-TOF mass spectrometry to establish both degree of purity and authenticity of structure.

### 2.6. Minimal Inhibitory Concentration (MIC) Antimicrobial Assays

Minimal inhibitory concentrations (MICs) for synthetic peptides, balteatide, and sauvatide were assessed against the model strains of Gram-positive bacteria, Gram-negative bacteria, and yeast, used before for initial qualitative zonal growth inhibition assays. Each model microorganism was initially incubated in Mueller-Hinton Broth (MHB) for 16–20 h. Upon achieving their respective logarithmic growth phases, as measured by the optical density (OD) of media at 550 nm, the cultures were diluted to 1 × 10^6^ colony-forming units (cfu)/mL for the bacteria and to 5 × 10^5^ cfu/mL for the yeast. Samples of these were then added to 96-well microtiter plates and mixed with the peptides at a range of concentrations (1–512 mg/L). Positive controls were included using the cytolytic bee venom peptide, melittin, and ampicillin, in a similar concentration range to the test peptides. After 24 h incubation, the OD of each well was measured at 550 nm using a Synergy HT plate reader (BioTek, USA), and the data were analysed using Graph Pad Prism 5 software. The MIC was defined as the minimum concentration of peptide that resulted in an OD that was the same as the negative control.

### 2.7. Haemolysis Assay

A 2% (v/v) suspension of red blood cells was prepared from defibrinated horse blood (TCS Biosciences Ltd., UK). Peptide solutions at different concentrations were prepared as described in the previous section. Red blood cell suspension samples (200 *μ*L) were incubated with a range of peptide concentrations (1–512 mg/L) as employed for antimicrobial MIC assays, at 37°C for 60 min. Lysis of red cells was assessed by measurement of optical density at *λ* = 550 nm using an ELISA plate reader (Biolise BioTek EL808). Negative controls employed consisted of a 2% (v/v) red cell suspension and sodium phosphate-buffered saline (PBS) in equal volumes and positive controls consisted of a 2% (v/v) red cell suspension and an equal volume of PBS containing 2% (v/v) of the nonionic detergent, Triton X-100 (Sigma-Aldrich).

### 2.8. Rat Urinary Bladder Smooth Muscle Bioassay

Male Wistar rats (250–300 g) were euthanised by carbon dioxide asphyxiation followed by cervical dislocation under appropriate UK animal licences and in keeping with institutional ethical guidelines. The rats were placed dorsal surface down and the abdomen was opened by means of an incision along the midventral line and subcutaneous fat was carefully dissected. The exposed urinary bladder was removed from each rat, emptied of urine, and placed in ice-cold Krebs solution (118 mM NaCl, 4.7 mM KCl, 25 mM NaHCO_3_, 1.15 mM NaH_2_PO_4_, 2.5 mM CaCl_2_, 1.1 mM MgCl_2_, and 5.6 mM glucose), equilibrated with 95% O_2_, 5% CO_2_. Muscle strips, 2 mm × 10 mm, were dissected from the bladder under a dissection microscope. These were tied at each end with a fine silk ligature (0.2 mm) with one end subsequently attached to a fixed pin and the other to a transducer in a 2 mL organ bath containing Krebs solution at 37°C flowing at 2 mL/min with constant bubbling of 95% O_2_, 5% CO_2_. After a 20 min equilibration period, muscle strips were tested for viability using 60 mM KCl. Peptide solutions, ranging in concentration from 10^−11^ to 10^−5^ M, were made in Krebs buffer and were used to construct dose-response curves. These were added to the bladder muscle strips in increasing concentrations with 5 min washes and 5 min equilibration periods between each dose. Each peptide concentration was applied to a minimum of five muscle strips. Changes in tension of the bladder muscle strips were recorded and amplified through pressure transducers connected to a PowerLab System (AD Instruments Pty Ltd.). Data were analysed to obtain the mean and standard error of responses by Student's* t-*test and dose-response curves were constructed using a best-fit algorithm through the data analysis package provided. Responses were plotted as percentages of maximal contraction against final molar concentrations of peptide present in the organ baths.

## 3. Results 

### 3.1. Identification and Structural Analysis of Balteatide

**Figure 1:**
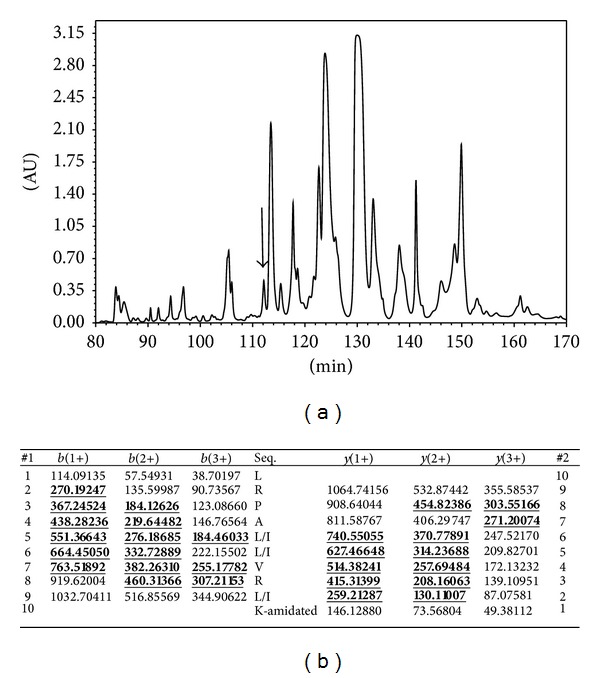
(a) Region of reverse phase HPLC chromatogram of* Phyllomedusa baltea* skin secretion indicating the absorbance peak corresponding to balteatide (arrow). The *Y*-axis represents the relative absorbance at *λ* 214 nm and the *X*-axis represents the retention time in minutes. (b) Predicted *b*- and *y*-ion series (singly, doubly, and triply charged) of balteatide. Ions observed in MS/MS spectra are indicated in bold typeface and are underlined.

Reverse-phase HPLC fractionation of* P. baltea* skin secretion resulted in a complex chromatogram. The elution position/retention time of the AMP-containing fraction (number 114), established by zonal growth inhibition assay, is indicated by an arrow (Figure 1(a)). MALDI-TOF analysis of a sample from this fraction revealed the presence of a major, singly charged peptide (M + H)^+^ of* m/z* 1178.5 (data not shown). Subsequent infusion of this fraction into an LCQ-Fleet electrospray ion-trap mass spectrometer and trapping of the predominant doubly charged (M + 2H)^2+^ ion at* m/z* 589.5, followed by MS/MS fragmentation, resulted in the generation of a sequence by the* de novo* sequencing software: L/I-R-P-A-L/I-L/I-V-R-I/L-K.amide (Figure 1(b)). This novel peptide was named balteatide, reflecting its species of origin and its structural similarity to a previously reported myotropic peptide named sauvatide, from the skin secretion of* Phyllomedusa sauvagei* [15].

### 3.2. Molecular Cloning of the cDNA Encoding the Biosynthetic Precursor of Balteatide

**Figure 2:**
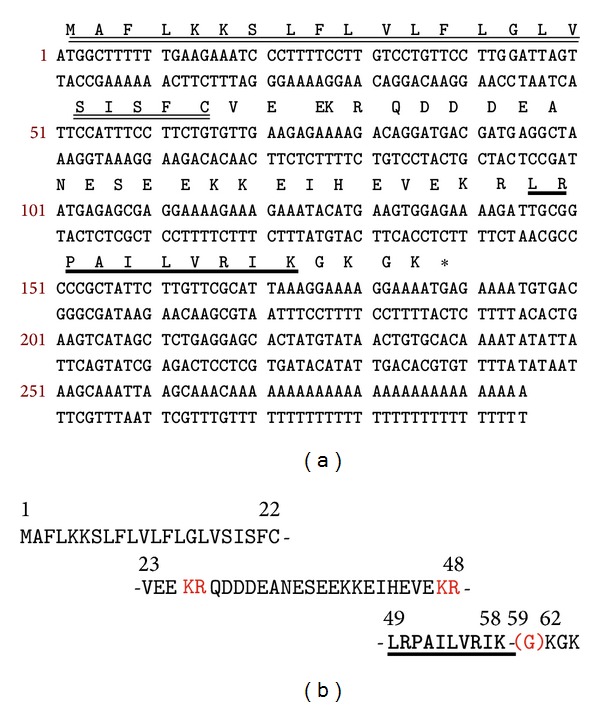
(a) Nucleotide and translated open-reading frame amino acid sequence of the sense strand of the cloned cDNA encoding the biosynthetic precursor of balteatide. The putative signal peptide is double-underlined and the mature balteatide sequence is single-underlined. The stop codon is indicated with an asterisk. (b) Domain architecture of the balteatide precursor. Residues 1–22 constitute the putative signal peptide. Residues 23–48 constitute the acidic spacer peptide region typified by classical-KR-(-Lys-Arg-) propeptide convertase processing sites (italicised and in bold typeface). The single copy of mature balteatide (residues 49–58) is underlined and in bold typeface and the C-terminal glycyl (G59) residue that donates the amide moiety is indicated in brackets.

**Figure 3:**
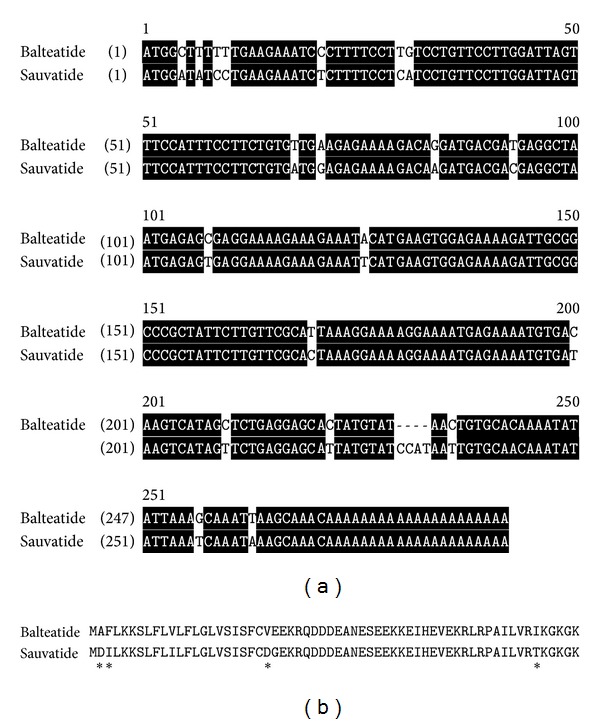
(a) Alignment of the nucleotide sequences of cDNAs encoding balteatide and sauvatide precursors. Identical bases in both are back-shaded in black. (b) Alignment of translated open-reading frame amino acid sequences of balteatide and sauvatide precursors. Sites of amino acid residue differences are indicated by asterisks.

A single cDNA encoding the biosynthetic precursor of balteatide was consistently cloned from the skin secretion-derived cDNA library using the RACE protocol described (Figure 2(a)). In terms of the balteatide precursor protein architecture, the N-terminal 22 amino acid residues encoded a putative signal peptide and the following 26 amino acid residues constituted an acidic amino acid residue-rich spacer peptide domain containing two classical-Lys-Arg-(-K-R-) propeptide convertase cleavage sites, the second of which immediately flanked the N-terminus of balteatide. The presence of an amide moiety on the C-terminal Lys (K) residue was implied by the presence of a strategically located Gly (G) residue that acts as an amide donor through the action of amidating enzyme complex. The C-terminal extension sequence, -KGK, was not present on the mature peptide identified in skin secretion and is presumably enzymatically removed after translation. The successful cloning of the balteatide precursor-encoding cDNA also facilitated resolution of the isobaric L/I residue ambiguity following MS/MS fragmentation sequencing and established the presence of L at position 1, I at position 5, L at position 6, and I at position 9 (Figures 2(a) and 2(b)). BLAST analysis of this sequence, using the National Center for Biotechnological Information (NCBI) online portal, revealed that the balteatide precursor displayed a 92% nucleotide sequence and a 90% amino acid sequence identity to the sauvatide precursor from the skin of the waxy monkey frog,* Phyllomedusa sauvagei* (Figures 3(a) and 3(b)). However, the respective mature peptides, balteatide and sauvatide, only differed in a single amino acid (I/T) at position 9.

### 3.3. Antimicrobial MIC Assays

**Figure 4:**
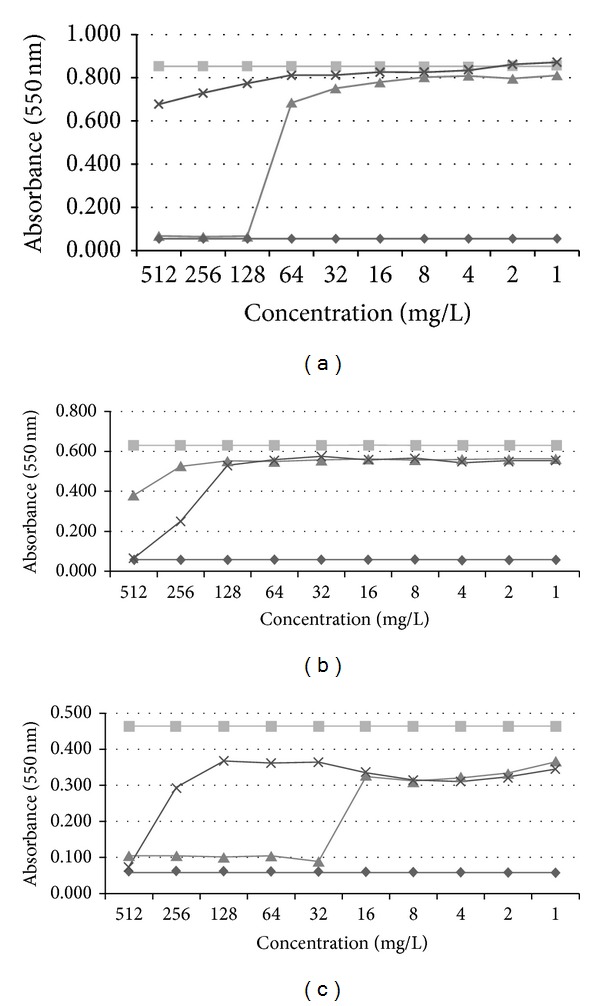
Dose-response curves of synthetic balteatide (▲) and sauvatide (×) with the three model test microorganisms: (a)* Escherichia coli* (NCTC 10418), (b)* Staphylococcus aureus* (NCTC 10788), and (c)* Candida albicans* (NCPF 1467).

The data derived from the parallel MIC determination experiments, using synthetic balteatide and sauvatide, are illustrated in Figures 4(a)–4(c). Balteatide was moderately active against* E. coli* with an MIC of 128 mg/L (109 *μ*M) and sauvatide was essentially ineffective at the highest concentration tested (512 mg/L; 439 *μ*M) (Figure 4(a)). Balteatide was essentially ineffective against* S. aureus* while sauvatide exhibited an MIC of 512 mg/L (439 *μ*M) (Figure 4(b)). The greatest difference in antimicrobial potency was observed with* C. albicans* against which balteatide exhibited an MIC of 32 mg/L (27 *μ*M) compared to that of sauvatide (512 mg/L; 439 *μ*M) (Figure 4(c)). All quantitative data, including the MICs obtained for the positive controls, are summarised in Table 1.

### 3.4. Haemolytic Activity Assay

**Figure 5:**
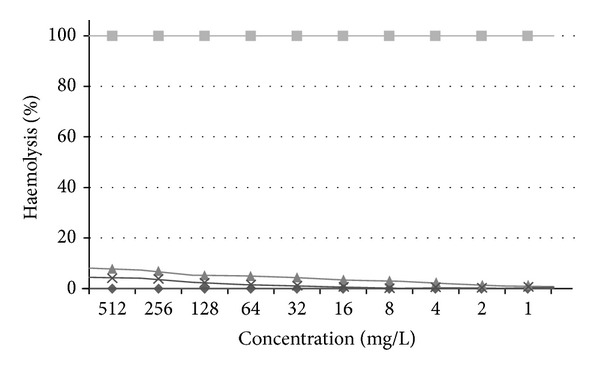
Haemolytic activity of synthetic balteatide (♦) and sauvatide (×), respectively. Positive (■) and negative (♦) controls are included for reference.

Both peptides were found to possess little haemolytic activity (less than 8%) at the highest concentrations tested (512 mg/L) (Figure 5). At the effective MICs for balteatide against* E. coli* (128 mg/L) and* C. albicans* (32 mg/L), the level of haemolytic activity was negligible.

### 3.5. Rat Urinary Bladder Smooth Muscle Pharmacology

**Figure 6:**
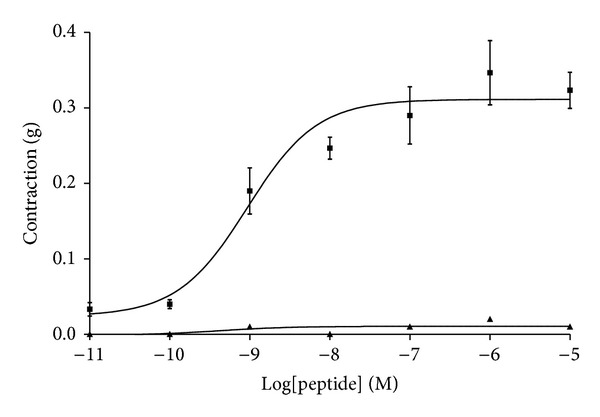
Dose-response curves of sauvatide (■) and balteatide (▲) on rat bladder smooth muscle preparations. Each point represents the mean and standard error of six determinations.

In this bioassay, the activities of both peptides were quite disparate despite their highly conserved primary structures (Figure 6). Sauvatide was a potent myotropic agent in rat urinary bladder smooth muscle with an EC_50_ of 4.7 nM (4.7 × 10^−9^ M). In contrast, balteatide was inactive in this smooth muscle preparation at concentrations up to 10 *μ*M (1 × 10^−5^ M).

## 4. Discussion

The spread of microbial resistance is leading to a pandemic in untreatable or difficult-to-treat infections of both humans and livestock [1–3, 16]. This resistance poses a massive threat to human health and food supplies and it is imperative that both academic and industrial bioscientists join forces in the quest for novel molecular solutions to this rapidly growing global problem [17]. Our increasing knowledge and database of naturally occurring antimicrobial peptides, whose origins in the biosphere are undoubtedly ancient, provide one possible avenue to pursue towards the goal of discovering or developing novel anti-infection therapeutics [2, 3, 18]. AMPs are now known to constitute a fundamental component of innate immunity/molecular defence across most forms of life, including bacteria [5–11]. AMPs tend to be potent and broad spectrum in action and can kill both Gram-negative and Gram-positive bacteria, including those strains which are resistant to conventional antibiotics. Amphibian skin secretions appear to be one of the richest natural sources of diverse antimicrobial peptides [12, 13, 19]. These peptides have been found to have efficacy against many Gram-negative and Gram-positive bacteria, fungi, protozoans, and some viruses, including HIV. They are thought to be particularly abundant in frog skin secretions as a defence against surface microbial colonisation, a consequence of both life forms being particularly abundant in the same biotopes [5, 6, 20, 21].

Here, we have described the identification and structural characterisation of a novel AMP, named balteatide, from the skin secretion of wild Peruvian purple-sided leaf frogs,* Phyllomedusa baltea.* This C-terminally amidated decapeptide is representative of a novel class of AMPs, a group of peptides which are particularly abundant and structurally diverse in phyllomedusine leaf frogs [12, 13]. This is the smallest of such peptides reported thus far from this source. It is generally agreed that amphibian skin AMPs, in common with those from many other sources, act in a membranolytic fashion to destroy the integrity of microorganisms [5–11]. To achieve this biological endpoint, there appears to be an optimum peptide chain length required to effectively span the membrane and this is considered to be considerably longer than ten residues [5–11, 21]. However, in keeping with some recent evidence that suggests that certain AMPs may act through other targets, some of which are intracellular [5–11, 20, 21], the possibility exists that balteatide may have such an alternative target. Its spectrum of antimicrobial action was found to be unusual in that it was most potent at inhibiting the growth of the yeast,* C. albicans*, less potent against the Gram-negative bacterium,* E. coli*, and least effective against the Gram-positive bacterium,* S. aureus*. Most AMPs are cationic and amphipathic in nature and are thought to initially bind electrostatically to the negatively charged phospholipids within prokaryotic membranes. A typical spectrum of decreasing efficacy for amphibian skin AMPs would be* S. aureus* >* E. coli* >* C. albicans*. Eukaryotic membranes, such as those in the yeast,* C. albicans*, are more resistant to the effects of these AMPs as a consequence of membrane compositional differences that provide a less anionic surface for cationic peptide interaction [6–11, 19, 22]. The apparent degree of specificity exhibited by balteatide for the eukaryotic test microorganism in the present study may imply a nonmembranolytic mode of action. This suggestion is provided with some credence by the lack of haemolytic activity exhibited by balteatide in the present study. Although many other amphibian skin AMPs may have higher potencies and spectra of actions, the apparent high degree of specificity of balteatide for the yeast demonstrated here is a unique attribute among these. Thus the preliminary observations reported here may provide a rationale for instigating more in-depth studies on balteatide with respect to its mode of action and molecular target. If this peptide should prove, in further studies, to be active against other types of fungi, it could represent the lead compound for the development of a novel class of antifungal therapeutic. If not, then it could be therapeutically useful against* C. albicans* infections which are in themselves sufficiently widespread and troublesome.

Following unequivocal establishment of the primary structure of balteatide, an NCBI BLAST search was performed which indicated that it was 90% identical to the myotropic peptide, sauvatide, previously isolated from the skin secretion of the waxy monkey frog,* Phyllomedusa sauvagei* [15]. Both peptides differed by just one amino acid residue at position 9 (Ile (I) in balteatide and Thr (T) in sauvatide). For this reason, sauvatide was chemically synthesised and subjected to antimicrobial and urinary bladder smooth muscle bioassays in parallel with balteatide. Sauvatide displayed little or no antimicrobial activity at the concentrations tested and was considerably less effective than balteatide in inhibiting the growth of* C. albicans* (439 *μ*M compared to 27 *μ*M). However, in the rat urinary bladder smooth muscle bioassay, sauvatide contracted the preparation with an EC_50_ of 4.7 nM (4.7 × 10^−9^ M) and balteatide was found to be completely ineffective up to a concentration of 10 *μ*M (1 × 10^−5^ M). These radical discrepancies in biological activities were surprising in view of the high degree of structural similarity between both peptides. The amino acid residue at position 9 thus appeared to be an essential determinant for both bioactivities. The EC_50_ value obtained for sauvatide in the smooth muscle assay is of an appropriate magnitude for a highly specific interaction with a discrete target receptor and this interaction appears from the current data to be highly dependent on the presence of a hydrophilic Thr residue at position 9 in the peptide chain. Substitution of this site with a hydrophobic Ile residue completely abolishes this activity. In contrast, this single hydrophobic Ile residue at this site appears to significantly enhance the peptides growth inhibitory action on the yeast. Many studies relating to studying the primary structural diversity in amphibian skin peptide families are considered mundane, but the current data illustrate quite unequivocally that even single residue changes in decapeptides can drastically alter their biological targeting and hence pharmacological profiles. Thus elucidation of the finer structural features of naturally occurring peptide families and screening for biological actions can often provide invaluable information about their structure/function requirements. When constructing a combinatorial library of a core lead peptide structure for drug development endpoints, this information could potentially provide prior warning about potential side effects and explanations for subsequently observed off-target effects. Many studies on the plethora of bradykinins and related peptides found in amphibian skin have previously illustrated this phenomenon as some single amino acid substitutions in the core nonapeptide sequence and short extensions to N- and C-terminals can change the ligand from a receptor agonist to antagonist [23–26].

In conclusion, the study of the structural diversity within discrete families of natural bioactive peptides, particularly those that are homologues of endogenous mammalian regulatory peptides, can provide libraries of unique agonists and antagonists for exploring the structure/activity requirements of endogenous receptors as an aid to rational analogue design. In addition, as many of the structural modifications observed in discrete members of these natural peptide families are counterintuitive, they have much to teach the pharmaceutical peptide engineer about peptide-based drug design. The targeting of sauvatide to putative receptors in urinary bladder smooth muscle and the effect of balteatide on inhibition of the growth of pathogenic yeast elegantly illustrate these suggestions and open up opportunities for further studies on both peptides as leads for both therapeutic applications.

## Figures and Tables

**Table 1:** Mean inhibitory concentrations (MICs) of each antimicrobial agent against the three model test microorganisms employed.

	MICs (mg/L and *μ*M)		
	*E. coli *	*S. aureus *	*C. albicans *
Balteatide	128 mg/L (109 *μ*M)	>512 mg/L (435 *μ*M)	32 mg/L (27 *μ*M)
Sauvatide	>512 mg/L (439 *μ*M)	512 mg/L (439 *μ*M)	512 mg/L (439 *μ*M)
Melittin	16 mg/L (6 *μ*M)	8 mg/L (3 *μ*M)	8 mg/L (3 *μ*M)
Ampicillin	8 mg/L (23 *μ*M)	0.0625 mg/L (0.2 *μ*M)	NE

NE: not effective.

## Data Deposition

The nucleotide sequence of Balteatide, from the skin secretion of* Phyllomedusa baltea*, has been deposited in the EMBL Nucleotide Sequence Database under the accession code HG937610.
